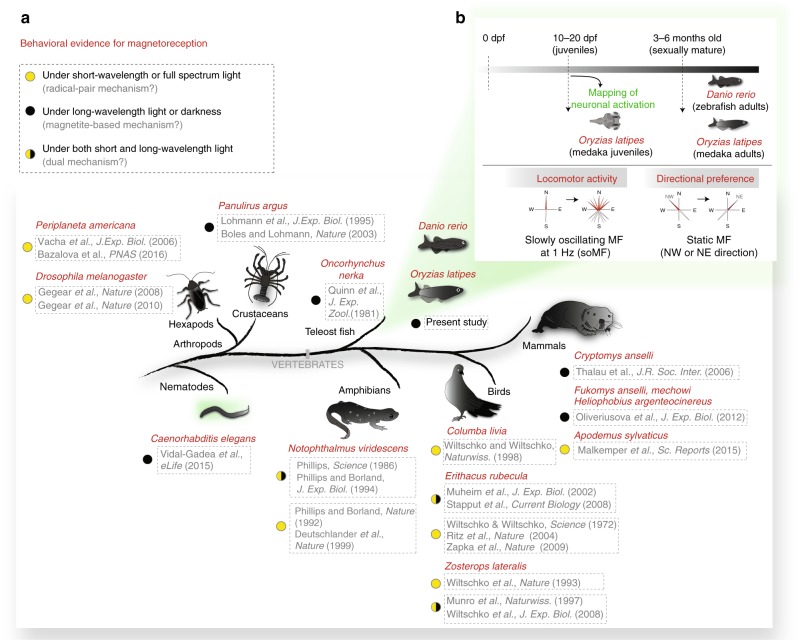# Author Correction: Zebrafish and medaka offer insights into the neurobehavioral correlates of vertebrate magnetoreception

**DOI:** 10.1038/s41467-018-05323-0

**Published:** 2018-07-17

**Authors:** Ahne Myklatun, Antonella Lauri, Stephan H. K. Eder, Michele Cappetta, Denis Shcherbakov, Wolfgang Wurst, Michael Winklhofer, Gil G. Westmeyer

**Affiliations:** 1 Institute of Biological and Medical Imaging, Helmholtz Zentrum München, Ingolstädter Landstrasse 1, 85764 Neuherberg, Germany; 2 Institute of Developmental Genetics, Helmholtz Zentrum München, Ingolstädter Landstrasse 1, 85764 Neuherberg, Germany; 3 Department of Nuclear Medicine, Technical University of Munich, Ismaninger Strasse 22, 81675 Munich, Germany; 4 Department of Earth- and Environmental Sciences Section Geophysics, Ludwig Maximilian University of Munich, Theresienstrasse 41, 80333 Munich, Germany; 5 Institute of Zoology 220, University of Hohenheim, 70593 Stuttgart, Germany; 6 Institute for Biology and Environmental Sciences IBU, Carl von Ossietzky University of Oldenburg, Carl-von-Ossietzky-Strasse 9-11, 26129 Oldenburg, Germany; 7 Research Center Neurosensory Science, Carl von Ossietzky Universität Oldenburg, D-26111 Oldenburg, Germany

Correction to: *Nature Communications*; 10.1038/s41467-018-03090-6; published online 23 February 2018

In the original version of this Article, *Oryzias latipes* was incorrectly spelt *Oryzias lapites* in the main text and in Fig. 1. These errors have been corrected in both the PDF and HTML versions of the Article.

**Figure 1:**