# Supplementary material for: Zebrafish and medaka offer insights into the neurobehavioral correlates of vertebrate magnetoreception
Source: Nat Commun. 2018 Feb 23;9:802. doi: 10.1038/s41467-018-03090-6 (PMC5824813; doi:10.1038/s41467-018-03090-6)
Supplement: Supplementary file 1 — Supplementary Information [file 41467_2018_3090_MOESM1_ESM.pdf]

Zebrafish and medaka offer insights into the neurobehavioral correlates of vertebrate magnetoreception

Myklatun, Lauri *et al.*

Supplementary Information

Table of Contents **Page**

Sup. Fig. 1: Deflection of the horizontal component of the geomagnetic field (GMF) 3

Sup. Fig. 2: Example analyses of zebrafish bearing (BE) in the directional preference assay 4

Sup. Fig. 3: Directional preference of zebrafish tested with no deflection (0°) of the magnetic field between two consecutive trials 5

Sup. Fig. 4: Directional preference of zebrafish tested under IR illumination with 90° deflection of the magnetic field but without preadaptation of the fish in darkness 6

Sup. Fig. 5: Analysis of thigmotaxis behavior in zebrafish under white light (WL) and infrared (IR) illumination 7

Sup. Fig. 6: Analysis of looping behavior in medaka under white light (WL) and infrared (IR) illumination 8

Sup. Fig. 7: Example analyses of medaka spatial preference (SP) in the directional preference assay 9

Sup. Fig. 8: Directional preference of medaka tested under IR illumination with 90° deflection of the magnetic field but without preadaptation of the fish in darkness 10

Sup. Fig. 9: Directional preference across groups of zebrafish and medaka 11

Sup. Fig. 10: Analysis of locomotor activity in zebrafish and medaka in response to stimulation with soMF 12

Sup. Fig. 11: Neuronal activation of the lateral hindbrain during stimulation with soMF in an independent cohort of juvenile medaka 13

Sup. Fig. 12: Technical drawings of the custom-built behavioral set-up used for the experiments with juveniles 14

Table of Contents continued

	Page
**Sup. Table 1**: Statistical analysis of different swimming behaviors	**15**
**Sup. Table 2**: Overview of the cohorts and experimental conditions of the directional preference assay	**15**
**Sup. Table 3**: Overview of the statistical tests run on the behavioral data from the directional preference assay	**15**
**Sup. Table 4**: Overview of the experimental conditions of the locomotor assay	**16**
**Sup. Table 5**: Overview of the statistical tests run on the behavioral data from medaka in the locomotor assay	**16**
**Sup. Table 6**: Overview of the statistical tests run on the behavioral data from zebrafish in the locomotor assay	**16**
**Sup. Table 7:** Overview of the statistical tests run on the pERK data from juvenile medaka brains	**17**

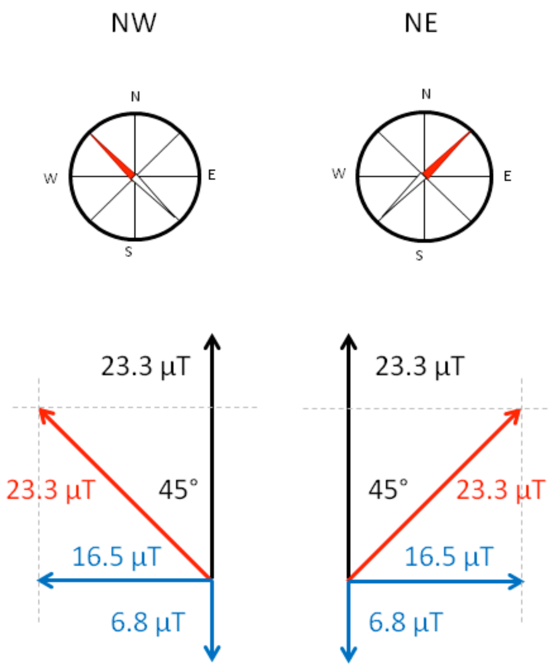

Supplementary Figure 1 | Deflection of the horizontal component of the geomagnetic field (GMF). The direction of the horizontal component of the GMF (black arrows) was deflected 45° by applying a magnetic field (MF) using the Helmholtz coils in the West-East orientation (blue). Another MF was applied in the South direction (blue) in order to generate a resulting magnetic field (red) equal in intensity to the ambient field.

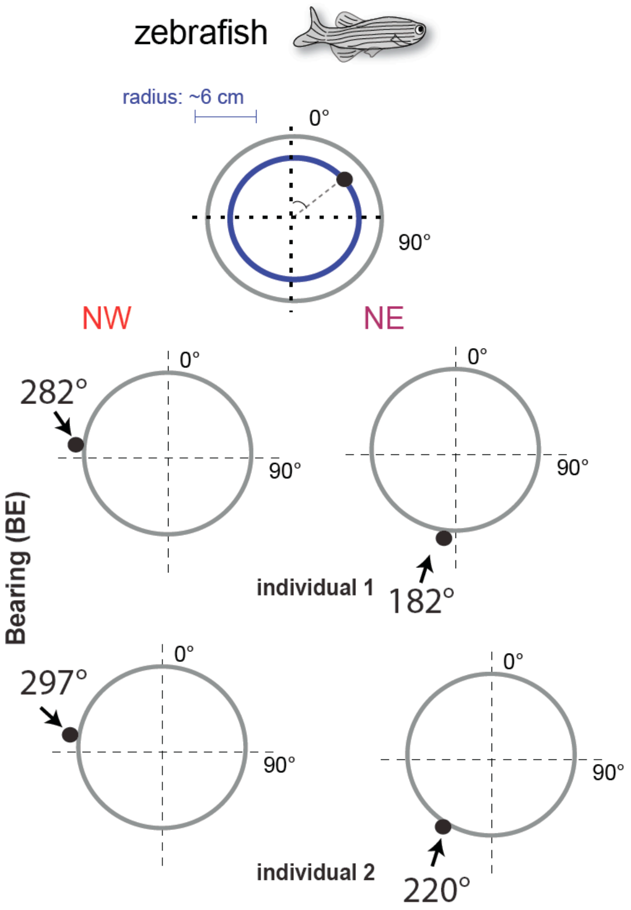

Supplementary Figure 2 | Example analyses of zebrafish bearing (BE) in the directional preference assay. For zebrafish, BE is calculated at 6 cm radius. BE is defined as the line between the center of the arena and the point where the fish first crossed a virtual circle (blue). Single bearing positions (black dots, arrows and angle) for two pairs of zebrafish individuals in NW (left) and NE (right) magnetic condition. The angular difference between the two bearings in NE and NW is then calculated (NE-NW).

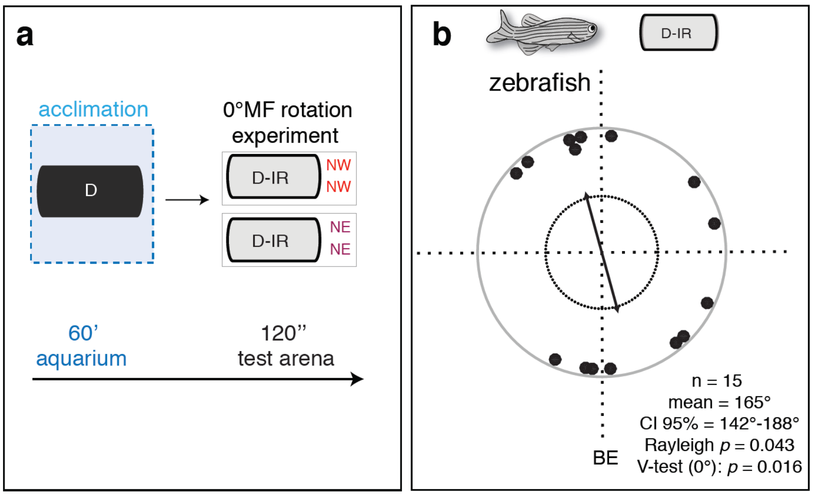

Supplementary Figure 3 | Directional preference of zebrafish tested with no deflection (0°) of the magnetic field between two consecutive trials. a: Schematic of the directional preference control experiment, where no deflection of the magnetic field (MF) was applied between consecutive trials (the fish were tested twice in NW or twice in NE). Fish were pre-adapted to darkness (D) and tested under IR illumination. **b:** The angular difference between the two trials was calculated from the bearing (BE) of the first trial subtracted from that of the second trial. Each dot in the circular plot represents the individual angular difference, the double arrow indicates axial symmetry of the mean vector computed by doubling the angles. The number of fish, the mean angle with the 95% confidence interval (CI), and the *p*-values are given for the Rayleigh test for circular uniformity as well as the V-test testing for a uniform distribution against the alternative hypothesis of angular differences with a mean of 0°.

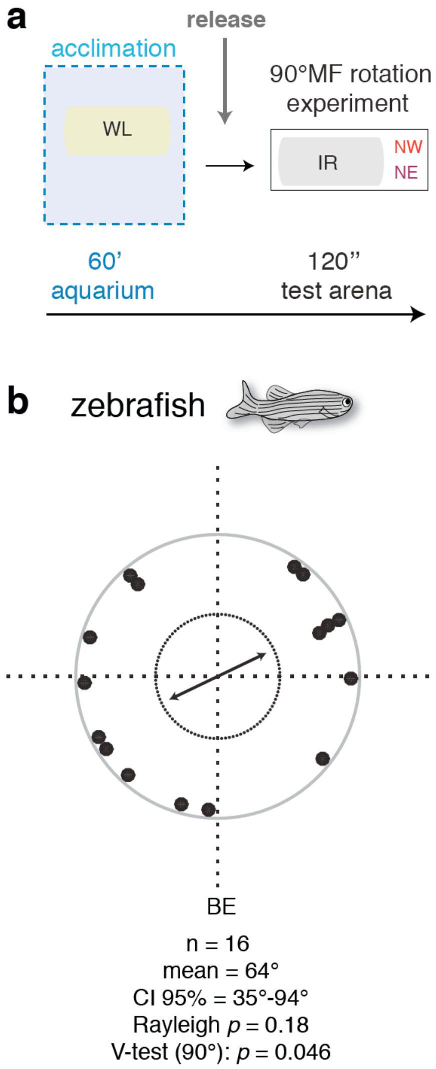

Supplementary Figure 4 | Directional preference of zebrafish tested under IR illumination with 90° deflection of the magnetic field but without preadaptation of the fish in darkness. a: Schematic of the directional preference assay with 90° horizontal deflection of the magnetic field (MF), between the NW and NE conditions. Fish are pre-adapted individually in white light (WL) and tested under IR illumination. **b:** Directional preference assessed in zebrafish. The change in directional preference is calculated as the angular difference between the NE and the NW condition. Analysis of the bearing (BE) is reported. Each dot in the circular plots represents the individual angular difference, the double arrow indicates the mean vector with axial symmetry, computed by doubling the angles. The number of fish, the mean angle with 95% confidence interval (CI), and the *p*-values for the Rayleigh as well as the V-test (testing for a uniform distribution against the alternative hypothesis of angular differences with a mean 90°) are reported under the circular plots.

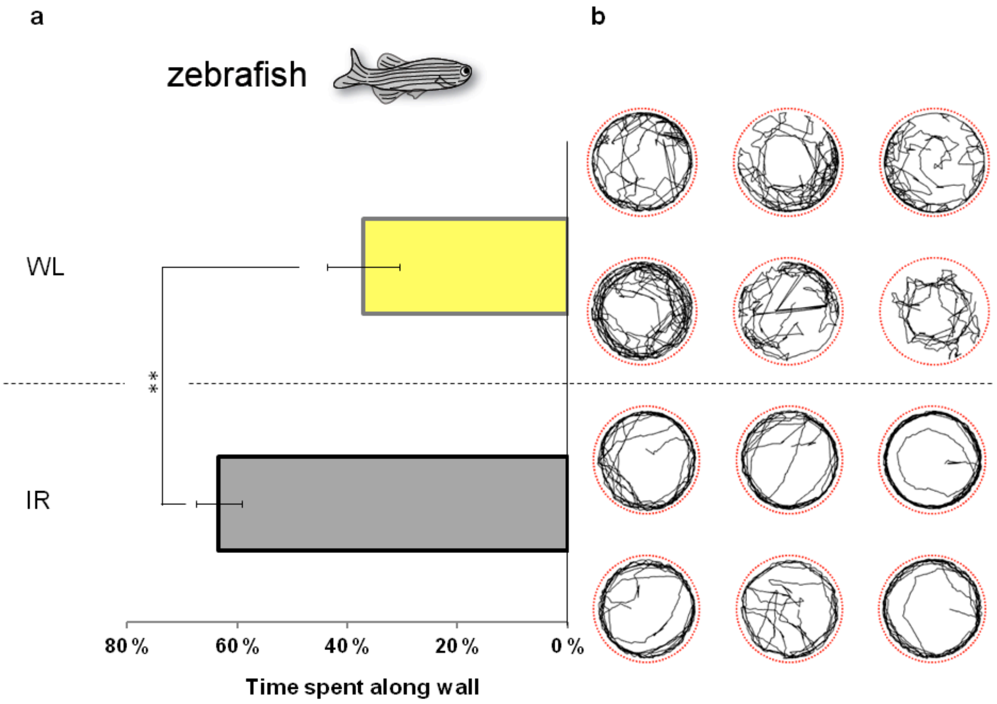

Supplementary Figure 5 | Analysis of thigmotaxis behavior in zebrafish under white light (WL) and infrared (IR) illumination. a: A significant increase in thigmotaxis was observed for the fish exploring the arena in IR compared to WL (n = 16, t-test: $p = 0.0016$, 2 tails). Thigmotaxis was defined as swimming closer to the wall than 2.5 cm. The bar graph represents the mean time spent along the wall for the different illuminations (WL, IR). The analysis was performed for the first minute of exploration and the first time a fish was introduced in the arena, independently of magnetic condition (NW, NE). Mean ± SEM are shown. **b:** Representative tracks of zebrafish individuals introduced to the unfamiliar arena either under WL (top) or IR illumination (bottom). For the purpose of showing examples, 6 tracks were randomly selected using a custom Matlab routine.

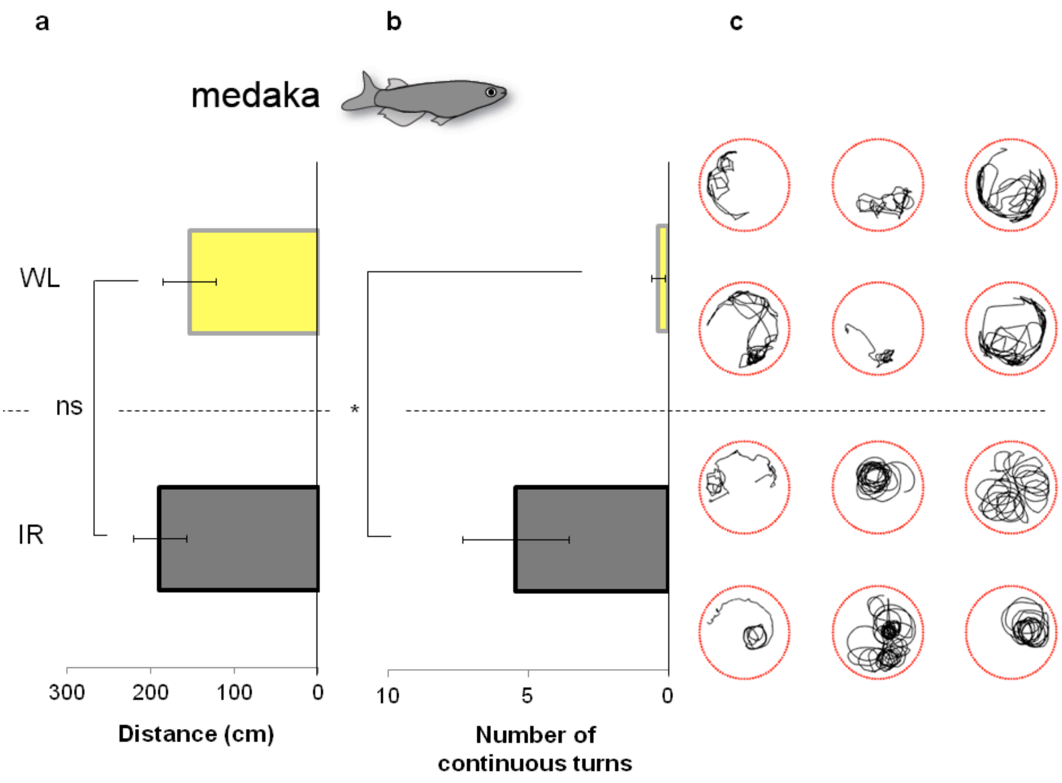

Supplementary Figure 6 | Analysis of looping behavior in medaka under white light (WL) and infrared (IR) illumination. a: the distance traveled by medaka (Cab) did not significantly differ between the two types of illumination (WL and IR). 154.3 ± 31.4 cm and 189.8 ± 31.9 cm were covered in distance respectively (n = 11, t-test: *p* = 0.36). **b**: In the IR condition, the fish showed an immediate looping behavior upon release in the circular arena. The frequency of continuous turns (looping) measured by the number of two consecutive complete turns (720° continuous turn), was significantly increased from 0.36 ± 0.25 when the fish were exploring the arena for two minutes with WL to 5.5 ± 1.9 with IR (n = 11, Wilcoxon signed rank test: *p* = 0.016). Mean ± SEM are shown. **c**: Representative tracks of medaka individuals introduced to the unfamiliar arena either with WL (top) or IR (bottom). For the plots in this figure, six swimming trajectories were randomly selected using a Matlab script.

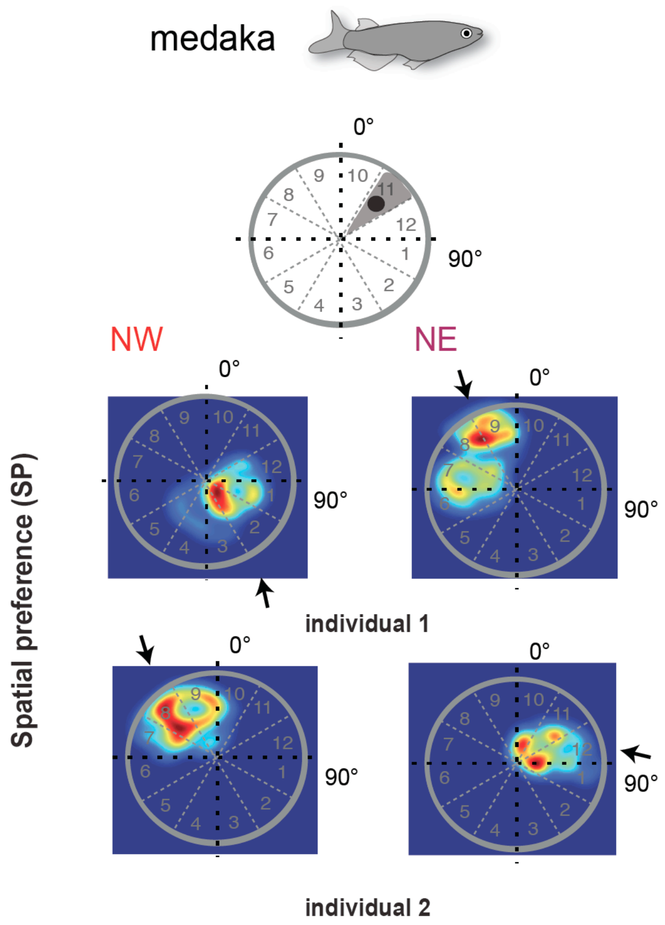

Supplementary Figure 7 | Example analyses of medaka spatial preference (SP) in the directional preference assay. The arena was virtually divided into 12 segments and the SP was calculated as the segment in which the fish spent most of the time (shaded gray segment). The heat maps show the position of two medaka individuals in the NW (left) and NE (right) magnetic conditions. The angular difference between the two most-visited segments in NE and NW is then calculated (NE-NW).

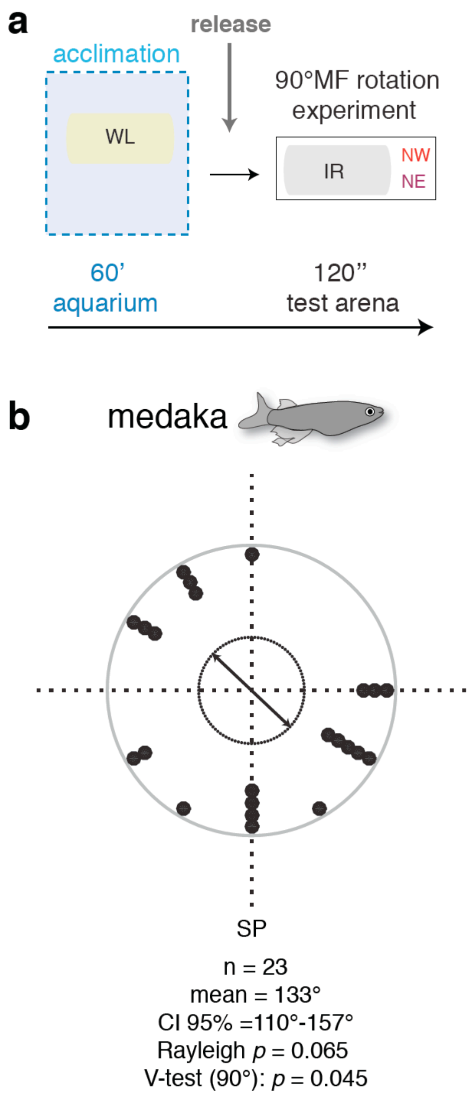

Supplementary Figure 8 | Directional preference of medaka tested under IR illumination with 90° deflection of the magnetic field but without preadaptation of the fish in darkness. a: schematics of the directional preference assay as in Supplementary Figure 4. **b:** Change in directional preference assessed in medaka with a 90° deflection of the magnetic field. Analysis of the spatial preference (SP) is reported. Each dot in the circular plots represents the individual angular difference between NE and NW, the double arrow indicates the mean vector with axial symmetry, computed by doubling the angles. The number of fish, the mean angle with 95% confidence interval (CI), and the *p*-values for the Rayleigh as well as the V-test for 90° are reported under the circular plots.

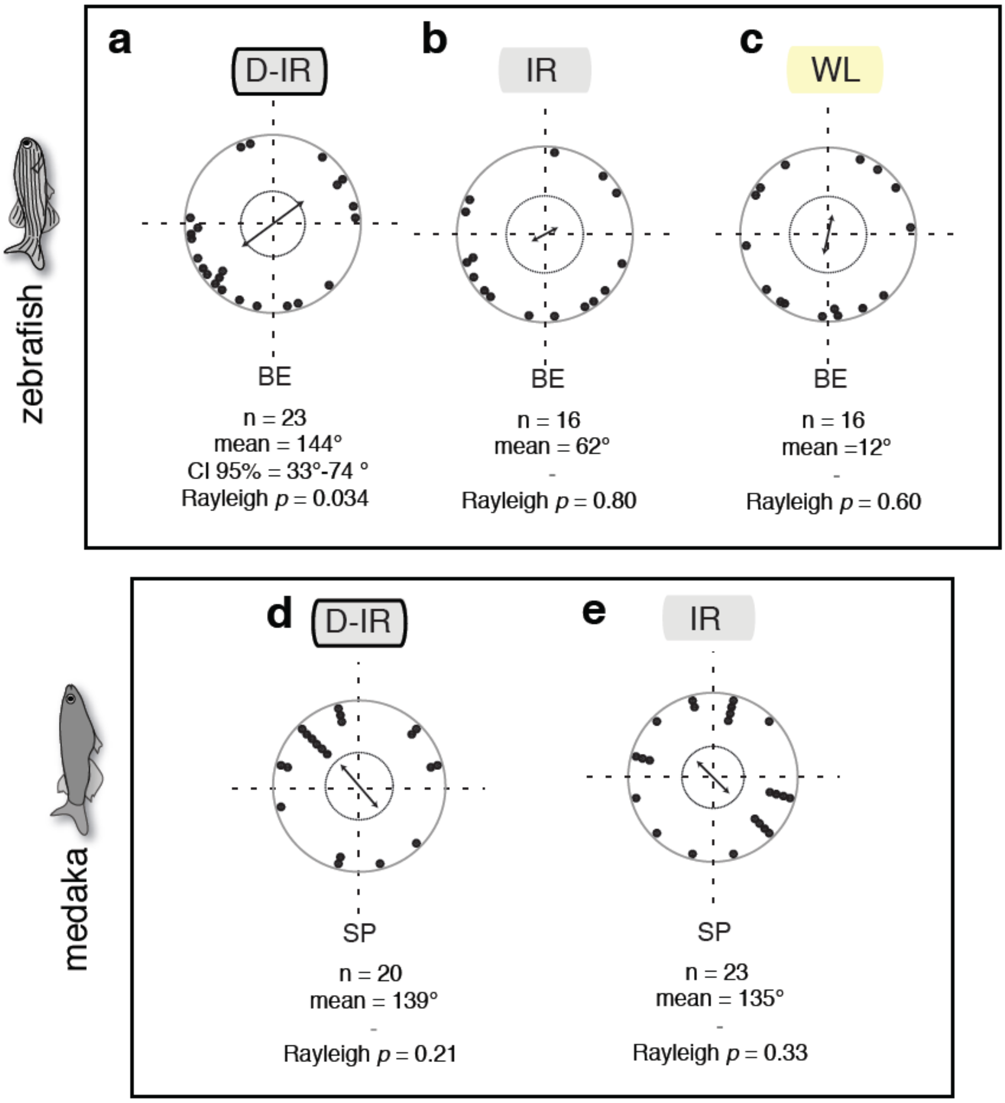

Supplementary Figure 9 | Directional preference across groups of zebrafish and medaka. Each dot in the circular plots represents the individual bearing (BE) for zebrafish (**a-c**) or spatial preference (SP) for medaka (**d,e**) during the first trial, after normalization to geomagnetic north (*i.e.* not the angular difference as in Fig. 2). The double arrow indicates the mean vector with axial symmetry, computed by doubling the angles. The number of fish, the mean angle and 95% confidence interval (CI), and *p*-value for the Rayleigh test are reported. The CI was not calculated for not significantly non-uniform distributions (b-e).

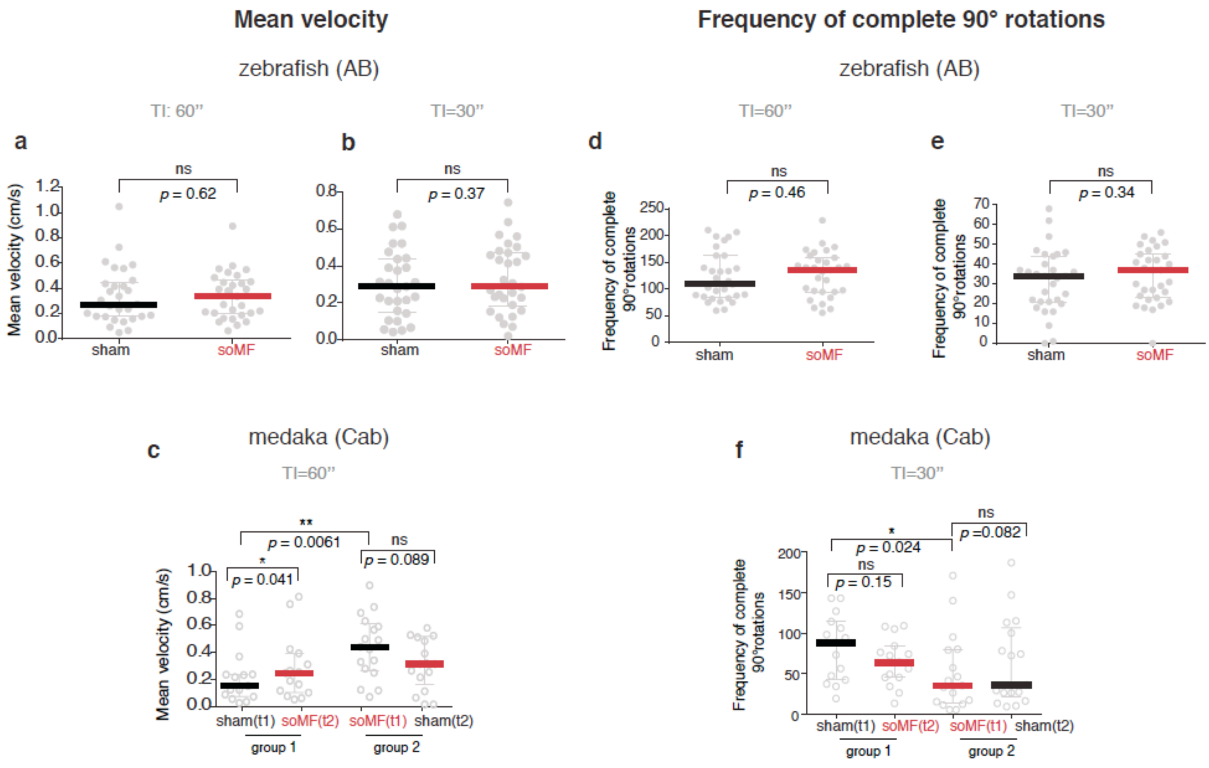

Supplementary Figure 10 | Analysis of locomotor activity in zebrafish and medaka in response to stimulation with soMF. a,b: In the locomotor assay schematized in Figure 3, juvenile zebrafish did not significantly change their swimming velocity during the soMF condition, neither for the time interval (TI) of 60 seconds (a), nor for 30 seconds (b) before and after the soMF. Single data points from the same condition (sham or soMF of group 1 and 2) were pooled and the median is shown together with the interquartile range, n = 29. Wilcoxon signed rank test, two tails. **c**: Plot showing the mean velocity for medaka of group 1 and group 2. Medaka significantly increased their velocity in soMF (soMF(t1) compared to sham(t1) and sham(t2)). Individuals tended to decrease their velocity in the sham condition (group 2). Plots showing the single data points for group1 and group 2, combined in Fig. 3b. Group 1: n = 15, Wilcoxon signed rank test, two tails. Group 2: n = 17, Wilcoxon signed rank test, two tails. Mann-Whitney test for control(t1) of group 1 and soMF(t1) of group 2, two tails. **d,e**: juvenile zebrafish did not significantly alter the frequency of complete 90° rotations, neither for the time interval (TI) of 60 seconds (d) nor for 30 seconds (e) before and after the soMF. Single data points from the same condition (sham or soMF of group 1 and 2) were pooled, and the median is shown together with the interquartile range, n = 30 (for a duration of 60 seconds), Wilcoxon signed rank test, two tails and n = 31 (30 second time interval), t-test, two tails. **f**: Plot showing the single data points for group 1 and group 2 combined in Fig. 3c for the frequency of complete 90° rotations in juvenile medaka. Juvenile medaka introduced in the arena under soMF (soMF(t1)) displayed a reduced frequency of rotations as compared to the sham control group (sham (t1)), n = 15 and n = 17 respectively, Mann-Whitney test, two tails. The frequency of rotations for individuals of the group 1 showed a downward trend under soMF. n = 15 Wilcoxon signed rank test, two tails. No clear trend is observed for group 2, n = 17, Wilcoxon signed rank test, two tails. All *p*-values are reported in the figure.

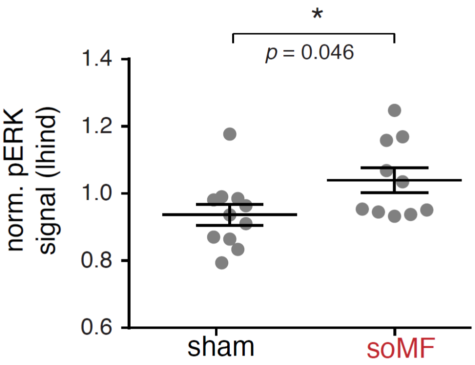

Supplementary Figure 11 | Neuronal activation of the lateral hindbrain during stimulation with soMF in an independent cohort of juvenile medaka. Quantifications of the normalized pERK signals (expressed as pERK/tERK) in the lateral hindbrain (lhind) of a second clutch of individuals (in addition to that shown in Fig. 4e) exposed to either the sham condition (control) or to soMF. The plot shows mean and standard error (± SEM). The *p*-value from the Welch's *t*-test unpaired t-test was used. Sham: n = 11, soMF: n = 10.

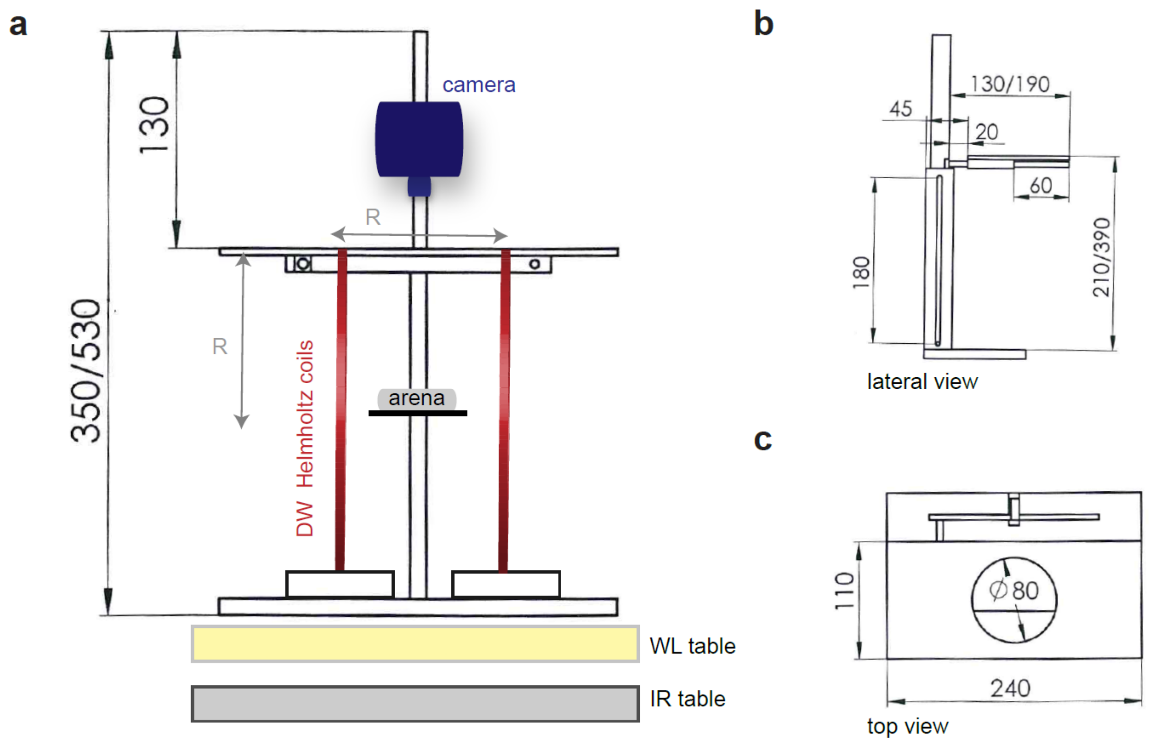

Supplementary Figure 12 | Technical drawings of the custom-built behavioral set-up used for the experiments with juveniles. a: The test arena was located in the center of a pair of double-wrapped Helmholtz coils (radius 12 cm, R). The set-up is made of transparent plastic such that a white light (WL) or infrared (IR) table for illumination can be placed on the bottom. Cameras can be screwed on the top of the set-up to record the behavior. The height and position of the arena and camera are adjustable. Lateral (**b**) and top (**c**) view of the set-up. All distances are indicated in millimeter.

Supplementary Table 1 | Statistical analysis of different swimming behaviors.

Fish species	Fish strain	Parameter	Condition	Statistical test	Pairing	DF	Tails	*n* WL	*n* IR	*p-value*	
Zebrafish	AB	thigmotaxis	WL - IR	t-test	unpaired	25	2	16	16	0.0016	**
Medaka	Cab	720° turns	WL - IR	Wilcoxon signed rank	paired	10	2	11	11	0.016	*
Medaka	Cab	distance	WL - IR	t-test	paired	10	2	11	11	0.36	ns

Supplementary Table 2 | Overview of the cohorts and experimental conditions of the directional preference assay.

					Illumination		Directional preference		
Fish species	Fish strain	# Fish	Age (months)	MF deflection	Acclimation	Test	Parameter	Angular difference	Group preferece
Zebrafish	AB	16	6	90	WL	WL	BE	* Rayleigh<br>** V-test	n.s<br>Rayleigh
Zebrafish	AB	16	6	90	WL	IR	BE	n.s<br>Rayleigh<br>* V-test	n.s<br>Rayleigh
Zebrafish	AB	24	12	90	Dark	IR	BE	* Rayleigh<br>* V-test	* Rayleigh
Zebrafish	AB	17	16	0	Dark	IR	BE	* Rayleigh<br>* V-test	n.s<br>Rayleigh
Medaka	CAB	24	4	90	WL	IR	SP	n.s<br>Rayleigh<br>* V-test	n.s<br>Rayleigh
Medaka	CAB	20	8	90	Dark	IR	SP	* Rayleigh<br>** V-test	n.s<br>Rayleigh

Supplementary Table 3 | Overview of the statistical tests run on the behavioral data from the directional preference assay.

Fish Species	Strain	Cohort	Illumination	MF deflection	Parameter	Distribution	*n*	R	Mean	CI 95%	Test	*p-value*	Test	*p-value*
Zebrafish	AB	1	WL	90	Ind. diff	axial	12	0.495	108	85-132	V-test(90)	0.010**	Rayleigh	0.049*
Zebrafish	AB	1	WL	-	cohort	axial	16	0.184	12	-	-	-	Rayleigh	0.59
Zebrafish	AB	1	IR	90	Ind. diff	axial	16	0.330	64	35-94	V-test(90)	0.046*	Rayleigh	0.18
Zebrafish	AB	1	IR	-	cohort	axial	16	0.121	62	-	-	-	Rayleigh	0.80
Zebrafish	AB	2	D-IR	90	Ind. diff	polar	24	0.347	125	79-170	V-test(90)	0.024*	Rayleigh	0.054
Zebrafish	AB	2	D-IR	-	cohort	axial	23	0.385	54	33-74	-	-	Rayleigh	0.031*
Zebrafish	AB	3	D-IR	0	Ind. diff	axial	15	0.453	165	142-188	V-test(0)	0.016*	Rayleigh	0.043*
Medaka	Cab	1	IR	90	Ind. diff	polar	23	0.343	133	110-156	V-test(90)	0.045*	Rayleigh	0.065
Medaka	Cab	1	IR	-	cohort	axial	23	0.220	135	-	-	-	Rayleigh	0.33
Medaka	Cab	2	D-IR	90	Ind. diff	axial	16	0.443	79	58-100	V-test(90)	0.006**	Rayleigh	0.041*
Medaka	Cab	2	D-IR	-	cohort	axial	20	0.282	139	-	-	-	Rayleigh	0.21

Supplementary Table 4 | Overview of the experimental conditions of the locomotor assay.

Fish species	Fish strain	# Fish	Age (dpf)	Illumination		Locomotor activity	
				Acclimation	Test	Mean velocity	Frequency of complete turns
Zebrafish	AB	31	>10	WL	WL	n.s	n.s
Medaka	CAB	32	>10	WL	WL	**	*

Supplementary Table 5 | Overview of the statistical tests run on the behavioral data from medaka in the locomotor assay.

Fish species	Fish strain	Parameter	Time interval of observation	Condition	Statistical test	Pairing	n sham	n soMF	*p-value*	
Medaka	Cab	Mean velocity	1 min	sham-soMF (t1+t2 pooled)	Wilcoxon signed rank test	paired	32	32	0.0057	**
Medaka	Cab	Mean velocity	1 min	sham(t1)-soMF(t2)	Wilcoxon signed rank test	paired	15	15	0.041	*
Medaka	Cab	Mean velocity	1 min	soMF(t1)-sham(t2)	Wilcoxon signed rank test	paired	17	17	0.089	ns
Medaka	Cab	Mean velocity	1 min	sham(t1)-soMF(t1)	Mann-Whitney test	unpaired	15	17	0.0061	**
Medaka	Cab	complete 90° turns	1 min	sham-soMF (t1+t2 pooled)	Wilcoxon signed rank test	paired	32	32	0.11	ns
Medaka	Cab	complete 90° turns	1 min	sham(t1)-soMF(t2)	Wilcoxon signed rank test	paired	16	16	0.044	*
Medaka	Cab	complete 90° turns	1 min	soMF(t1)-sham(t2)	Wilcoxon signed rank test	paired	17	17	0.79	ns
Medaka	Cab	complete 90° turns	1 min	sham(t1)-soMF(t1)	Mann-Whitney test	unpaired	15	17	0.10	ns
Medaka	Cab	complete 90° turns	30 s	sham-soMF (t1+t2 pooled)	Wilcoxon signed rank test	paired	32	32	0.033	*
Medaka	Cab	complete 90° turns	30 s	sham(t1)-soMF(t2)	Wilcoxon signed rank test	paired	15	15	0.15	ns
Medaka	Cab	complete 90° turns	30 s	soMF(t1)-sham(t2)	Wilcoxon signed rank test	paired	17	17	0.082	ns
Medaka	Cab	complete 90° turns	30 s	sham(t1)-soMF(t1)	Mann-Whitney test	unpaired	15	17	0.024	*

Supplementary Table 6 | Overview of the statistical tests run on the behavioral data from zebrafish in the locomotor assay.

Fish species	Fish strain	Parameter	Time interval of observation	Condition	Statistical test	Pairing	n sham	n soMF	*p-value*	
Zebrafish	AB	Mean velocity	1 min	sham-soMF (t1+t2 pooled)	Wilcoxon signed rank test	paired	29	29	0.62	ns
Zebrafish	AB	Mean velocity	1 min	sham(t1)-soMF(t2)	Wilcoxon signed rank test	paired	16	16	0.90	ns
Zebrafish	AB	Mean velocity	1 min	soMF(t1)-sham(t2)	Wilcoxon signed rank test	paired	14	14	0.26	ns
Zebrafish	AB	Mean velocity	1 min	sham(t1)-soMF(t1)	Mann-Whitney test	unpaired	16	14	0.70	ns
Zebrafish	AB	complete 90° turns	1 min	sham-soMF (t1+t2 pooled)	Wilcoxon signed rank test	paired	30	30	0.46	ns
Zebrafish	AB	complete 90° turns	1 min	sham(t1)-soMF(t2)	Wilcoxon signed rank test	paired	16	16	0.044	*
Zebrafish	AB	complete 90° turns	1 min	soMF(t1)-sham (t2)	Wilcoxon signed rank test	paired	14	14	0.79	ns
Zebrafish	AB	complete 90° turns	1 min	sham(t1)-soMF(t1)	Mann-Whitney test	unpaired	16	14	0.13	ns
Zebrafish	AB	Mean velocity	30 s	sham-soMF (t1+t2 pooled)	Welch's t-test	paired	29	29	0.37	ns
Zebrafish	AB	complete 90° turns	30 s	sham-soMF (t1+t2 pooled)	Welch's t-test	paired	31	31	0.34	ns
Zebrafish	AB	complete 90° turns	30 s	sham(t1)-oMF(t2)	Welch's t-test	paired	16	16	0.013	*
Zebrafish	AB	complete 90° turns	30 s	soMF(t1)-sham(t2)	Welch's t-test	paired	15	15	0.44	ns
Zebrafish	AB	complete 90° turns	30 s	sham(t1)-soMF(t1)	Welch's t-test	unpaired	29	29	0.13	ns

Supplementary Table 7 | Overview of the statistical tests run on the pERK data from juvenile medaka brains.

Anatomical region (sham-soMF)	Statistical test	Pairing	*n* sham	*n* soMF	*p*-value		Comments
Olfactory epithelium (oe)	Welch's t-test	unpaired	10	10	0.75	ns	FDR-adjusted
Pineal gland (pg)	Welch's t-test	unpaired	10	10	0.41	ns	FDR-adjusted
Left Habenula (lHb)	Welch's t-test	unpaired	9	10	0.21	ns	FDR-adjusted
Right Habenula (rHb)	Welch's t-test	unpaired	9	10	0.98	ns	FDR-adjusted
Lateral cerebellum (lcb)	Welch's t-test	unpaired	9	11	0.24	ns	FDR-adjusted
Lateral hindbrain (lhind)	Welch's t-test	unpaired	10	11	0.048	*	FDR-adjusted
Lateral hindbrain (lhind)	Welch's t-test	unpaired	11	10	0.046	*	
