# Supplementary material for: Zebrafish and medaka offer insights into the neurobehavioral correlates of vertebrate magnetoreception
Source: Nat Commun. 2018 Feb 23;9:802. doi: 10.1038/s41467-018-03090-6 (PMC5824813; doi:10.1038/s41467-018-03090-6)
Supplement: Supplementary file 3 — Description of Additional Supplementary Files [file 41467_2018_3090_MOESM3_ESM.pdf]

Description of Supplementary Files

File Name: Supplementary Movie 1
Description: **Swimming behaviour of zebrafish under white light (WL) and infrared (IR) illumination.** In the directional preference assay, zebrafish (AB strain) were tested either under white light (WL, left) or infrared illumination (IR, right). Only in the IR condition, the fish engaged in thigmotaxis after release from the center of the arena. The position of the fish was tracked with a routine written in Matlab; the background was subtracted for better visualization of the swimming trajectory.

File Name: Supplementary Movie 2
Description: **Swimming behaviour of medaka under white light (WL) and Infrared (IR) illumination.** Medaka (Cab strain) showed a different swimming behaviour while exploring a circular arena under white light (WL, left) compared to the looping behaviour they adopted under infrared illumination (IR, right). The video shows the same individual in both conditions for one minute (using a custom-written Matlab routine that subtracts the background for better visualization of the swimming trajectory).
